# Mannose binding lectin: a biomarker of systemic lupus erythematosus disease activity

**DOI:** 10.1186/ar4057

**Published:** 2012-10-15

**Authors:** Aditya K Panda, Jyoti R Parida, Rina Tripathy, Sarit S Pattanaik, Balachandran Ravindran, Bidyut K Das

**Affiliations:** 1 Infectious Disease Biology Group, Institute of Life Sciences, Nalco Square, Bhubaneswar, Odisha, 751023, India; 2 Department of Medicine, SCB Medical College, Mangalabag, Cuttack, Odisha, 753007, India; 3 Department of Biochemistry, SCB Medical College, Mangalabag, Cuttack, Odisha, 753007, India

## Abstract

**Introduction:**

A role for mannose binding lectin (MBL) in autoimmune diseases has been demonstrated earlier and elevated level of MBL has been shown in systemic lupus erythematosus (SLE) patients. In the current study, we investigated MBL as a potential biomarker for disease activity in SLE.

**Methods:**

In a case control study SLE patients (93 females) and 67 age, sex, ethnicity matched healthy controls were enrolled. Plasma MBL levels were quantified by enzyme linked immunosorbent assay (ELISA). Clinical, serological and other markers of disease activity (C3, C4 and anti-dsDNA) were measured by standard laboratory procedures.

**Results:**

Plasma MBL levels were significantly high in SLE patients compared to healthy controls (P < 0.0001). MBL levels were variable in different clinical categories of SLE. Lower levels were associated with musculoskeletal and cutaneous manifestations (P = 0.002), while higher and intermediate MBL levels were significantly associated with nephritis in combination with other systemic manifestations (P = 0.01 and P = 0.04 respectively). Plasma MBL correlated with systemic lupus erythematosus disease activity index (SLEDAI) (P = 0.0003, r = 0.36), anti-dsDNA (P < 0.0001, r = 0.54), proteinuria (P < 0.0001, r = 0.42) and negatively correlated with C3 (P = 0.007, r = -0.27) and C4 (P = 0.01, r = -0.29).

**Conclusions:**

Plasma MBL is a promising marker in the assessment of SLE disease activity.

## Introduction

Systemic lupus erythematosus (SLE) is a multi-factorial chronic autoimmune disorder characterized by dysfunction of T and B lymphocytes and affects various vital organ systems; 70 to 90% of SLE patients are female [1]. The etiology of the disease is still unclear, although environmental, host genetic and hormonal factors have been proposed to play major roles in pathogenesis of SLE [2]. There are limited studies on Indian SLE patients. Although the prevalence of SLE in India is rare (3/100,000) [3], the survival rates of these patients (70% at 5 years, 50% at 10 years) are very low compared to Western cohorts [4,5]. Several factors are thought to be responsible for poor survival rates, the most important being delayed diagnosis [6]. Several serological biomarkers such as complement components C3 and C4, and anti-dsDNA have been identified, which are not always consistent [7,8].

The innate immune system plays an important role in the pathogenesis of SLE [9]. Mannose binding lectin (MBL) is an essential component of innate immunity that recognizes carbohydrate residues on the surface of micro-organisms, namely, bacteria, viruses, fungi, and protozoa, and activates the complement system through MBL-associated serine proteases [10]. Upon activation, early complement factor C3 is cleaved to C3a and C3b. C3a induces inflammation and recruits phagocytes [11]. C3b enhances opsonisation by binding to both phagocytes and pathogens [12]. In addition, C3b binds to other proteins on the surface of pathogens, forming membrane attack complexes which clear foreign pathogens [13].

The role of MBL in the pathogenesis of SLE has so far not been clearly defined. Higher plasma MBL levels in SLE patients have been reported in various populations [14-16]. MBL appears to have a dual mode of action: increased MBL leads to enhanced complement activation and tissue damage, while its deficiency has been linked with defective clearance of apoptotic cells that provide a stimulus for autoantibody formation [17]. Also, MBL deficiency is linked to susceptibility to secondary infections, which is presumed to be a contributory factor in the development of SLE [18].

Variants of the MBL2 gene associated with lower plasma MBL levels have been associated with susceptibility to SLE [19-23] and the development of nephritis [19,20]. Recently, we have observed predisposition of patients with low MBL-producing genotype (LXA/LYB, LYB/LYB and LXB/LXB) to develop SLE and autoimmune hemolytic anemia (AIHA) [24]. Literature is limited on the association of MBL with disease activity [14,25]. The current study investigates the association between plasma MBL levels and SLE disease activity in a cohort of female patients from Eastern India.

## Materials and methods

### Patients and controls

Female patients attending the Rheumatology Clinic and/or admitted to the Department of Medicine of SCB Medical College, Cuttack, Odisha, India, were enrolled. SLE diagnosis was based on the revised American College of Rheumatology (ACR) classification criteria [26]. Various manifestations were categorized after detailed clinical examination and laboratory investigations (Table 1). Disease activity was assessed by the SLE Disease Activity Index (SLEDAI) and recorded. About 5 ml blood was collected from each patient. For each SLE patient, complete blood count (CBC), serum urea, serum creatinine, liver function test anti nuclear antibody (Hep2), antibodies to extractable nuclear antigen (ENA), anti- dsDNA antibody titers, complements (C3, C4), urinalysis and 24- hour proteinuria were analysed by standard procedure. Sixty-seven age- and ethnicity-matched unrelated healthy females were taken as healthy controls (HCs). The study was approved by the Institutional Ethical Committee of SCB Medical College Cuttack, Odisha, India. Informed written consent was obtained from each participant.

**Table 1:** Clinical characteristics of patients with systemic lupus erythematosus (SLE) and healthy controls (HC)

Clinical profiles	SLE (*n *= 93)	HC (*n *= 67)
Sex, male/female	0/93	0/67
Age, years, mean ± SD	28.80 ± 8.891	29.14 ± 6.48
Duration of disease, years, mean ± SD	1.927 ± 2.151	-
Disease onset, years, mean ± SD	25.78 ± 8.630	-
ACR criteria		
Photosensitivity	28 (30)	-
Malar rash	39 (42)	-
Discoid rash	15 (16)	-
Oral ulcer	47 (51)	-
Arthritis	49 (53)	-
NPSLE	26 (28)	-
Carditis	14 (15)	-
AIHA	10 (11)	-
Serositis	18 (19)	-
Nephritis	52 (56)	-
Pneumonitis	11 (12)	-

Data are number (%) of participants unless otherwise specified. ACR, American College of Rheumatology; NPSLE, neuropsychiatric SLE; AIHA, autoimmune hemolytic anemia.

### Plasma MBL measurement

MBL levels in plasma were quantified by ELISA, according to the manufacturer's instructions (R&D systems, Minneapolis, MN, USA). All samples and standards were measured in duplicate and concentrations were determined from a standard curve using mean optical density values. Serum MBL concentrations were expressed as μg/ml.

### Statistical analysis

Statistical analysis was performed using Graph Pad Prism, version 5.01 (Graph Pad Software, La Jolla, CA, USA). Mean MBL levels in HCs and SLE patients and mean values among different clinical categories were analyzed by the Student's *t*-test. The distribution of various MBL producer phenotypes (high, low and intermediate) in different clinical groups of SLE patients was analyzed by the Fisher exact test. Correlations among various parameters were evaluated with Spearman's rank correlation test. A *P*-value < 0.05 was considered significant.

## Results

### Plasma MBL levels in SLE patients and healthy controls

**Figure 1:**
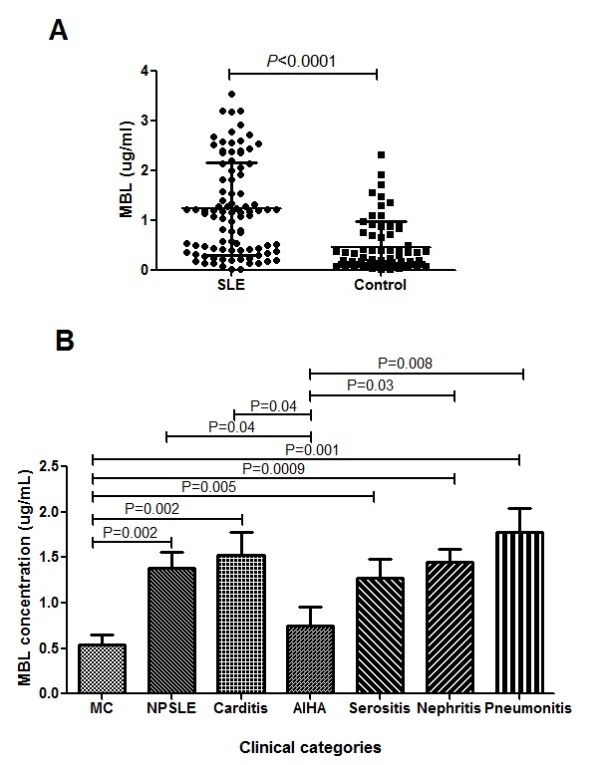
**Plasma mannose binding lectin (MBL) levels in systemic lupus erythematosus (SLE) patients and healthy controls (HCs)**. (**A**) Plasma samples from HCs (*n *= 67) and SLE patients (*n *= 93) were quantified by ELISA according to the manufacturer's instructions. SLE patients displayed significantly higher concentrations of MBL compared to HCs (*P *< 0.0001). (**B**) Based on clinical and biochemical parameters, SLE patients were further categorized as musculoskeletal or cutaneous (MC) (*n *= 16), neuropsychiatric systemic lupus erythematosus (NPSLE) (*n *= 26), carditis (*n *= 14), autoimmune hemolytic anemia (AHIA) (*n *= 10), serositis (*n *= 18), nephriris (*n *= 52), pneumonitis (*n *= 11) and MBL levels were compared among them. Dots represent individual samples; bars show the mean ± SD. The Student's *t*-test was used to compare MBL concentrations among clinical categories.

SLE patients displayed significantly higher levels of MBL compared to the HCs (*P *< 0.0001) (Figure 1A). Based on clinical and biochemical parameters, SLE patients were categorized into various clinical phenotypes and MBL levels were compared (Figure 1B). Patients with musculoskeletal and cutaneous manifestations displayed lower MBL levels compared to those with neuropsychiatric SLE (NPSLE) (*P *= 0.002), carditis (*P *= 0.002), serositis (*P *= 0.005), nephritis (*P *= 0.0009) and pneumonitis (*P *= 0.001). In addition, MBL levels were significantly low in AIHA when compared with NPSLE (*P *= 0.04), carditis (*P *= 0.04), nephritis (*P *= 0.03), pneumonitis (*P *= 0.008) cases.

Based on their plasma MBL levels (Figure 1A) we grouped the SLE patients arbitrarily into three categories: low (mean MBL 0.33 μg/ml), intermediate (mean MBL 1.03 μg/ml) and high (mean MBL 2.04 μg/ml). On further analyses, intermediate and high MBL levels were predominantly found in patients with nephritis in combination with other systemic involvement such as, carditis, serositis, and NPSLE. Most patients with musculoskeletal and cutaneous manifestations displayed low levels of MBL. (Table 2)

**Table 2:** Distribution of different mannose binding lectin (MBL) phenotypes in clinical categories of systemic lupus erythematosus (SLE)

Clinical categories	High MBL (*n *= 44)	Intermediate MBL (*n *= 13)	Low MBL (*n *= 36)	*P*-value; odds ratio
Nephritis	13 (29)	4 (31)	10 (28)	NS
Nephritis and carditis, or NPSLE, or serositis	16 (36)	5 (38)	4 (11)	0.01; 4.57^1^ 0.04; 5.00**^2^**
AIHA	4 (9)	3 (23)	3 (8)	NS
Carditis	5 (11)	0	1 (3)	NS
NPSLE	14 (31)	4 (31)	8 (22)	NS
Serositis	8 (18)	3 (23)	7 (19)	NS
Musculoskeletal and cutaneous only	2 (5)	3 (23)	11 (31)	0.002; 9.42^1^

^1^High vs. Low; **^2^**Intermediate vs. Low; data are number (%) of participants. Based on the plasma MBL levels, SLE patients were categorised as high MBL producers (mean 2.04 μg/ml), intermediate MBL producers (mean 1.03 μg/ml), or low MBL producers (mean 0.33 μg/ml). The Fisher exact test was used to compare the distribution of MBL phenotypes in SLE manifestations. AIHA, autoimmune hemolytic anemia; NPSLE, neuropsychiatric systemic lupus erythematosus; NS, not significant.

### MBL levels positively correlated with SLEDAI scores

**Figure 2:**
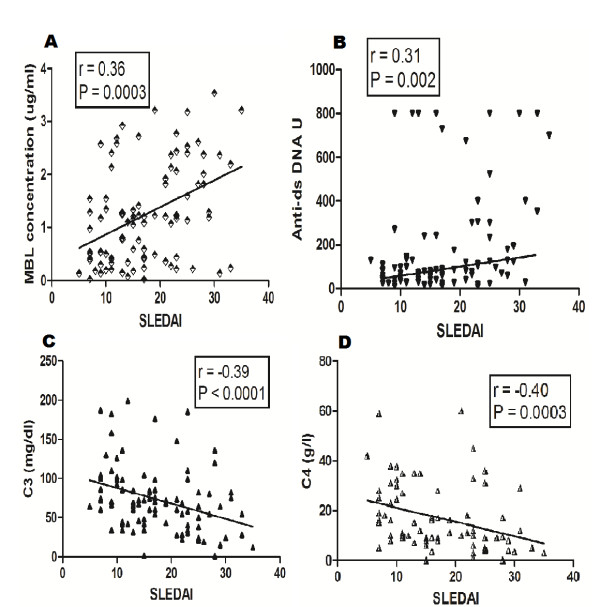
**Correlation of SLEDAI scores with plasma levels of mannose binding lectin (MBL), Anti-dsDNA, and complement components C3 and C4**. SLEDAI scores correlated positively with plasma MBL (**A**) and anti-dsDNA levels (**B**). In contrast, plasma C3 (**C**) and C4 (**D**) levels negatively correlated with the SLEDAI. Dots represent individual samples. Correlation analysis was performed using the Spearman correlation coefficient. *P *< 0.05 was considered significant.

Analysis of data in SLE patients revealed a significant positive correlation between plasma MBL levels and the SLEDAI scores as shown in Figure 2A (*P *= 0.0003, *r *= 0.36). Other investigators have reported an association of plasma parameters, such as anti-dsDNA, C3 and C4, with disease activity. Our findings on plasma levels of anti-dsDNA, C3 and C4 and their correlation with the SLEDAI scores (Figure 2B, C and 2D) are in conformity to earlier reports [27,28]. The SLEDAI scores correlated positively with dsDNA antibody levels (*P *= 0.002, *r *= 0.31), while it correlated negatively with C3 (*P *< 0.0001, *r *= -0.39) and C4 (P = 0.0003, *r *= -0.40) respectively.

### Correlation of MBL with anti-dsDNA, C3 and C4

**Figure 3:**
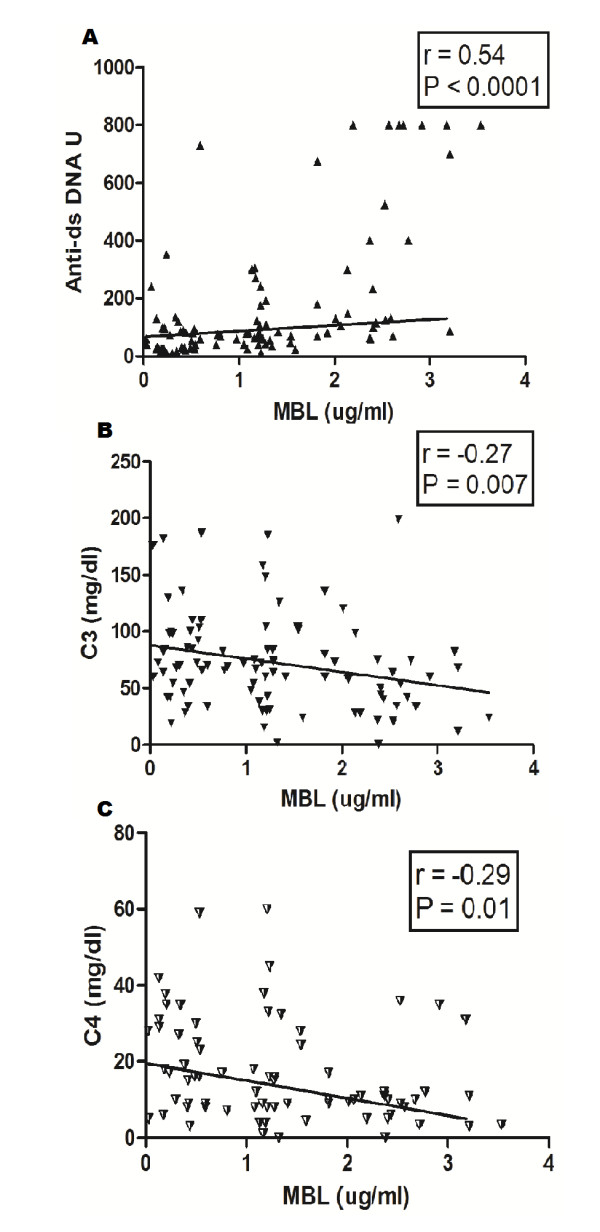
**Correlation of plasma mannose binding lectin (MBL) levels with anti-dsDNA, and complement components C3 and C4**. Plasma MBL levels correlated positively with anti-dsDNA (**A**) and negatively with plasma levels of C3 (**B**) and C4 (**C**). Dots represent individual samples. Correlation analysis was performed using the Spearman correlation coefficient. *P *< 0.05 was considered significant.

We further analysed correlations between MBL levels with the disease markers such as anti-dsDNA, C3, and C4 and the results are shown in Figure 3. A positive correlation was observed with anti-dsDNA (*P *< 0.0001, *r *= 0.54) and a negative correlation with C3 and C4 (*P *= 0.007, *r *= -0.27 and *P *= 0.01, *r *= -0.29 respectively).

### Plasma MBL level is associated with proteinuria

**Figure 4:**
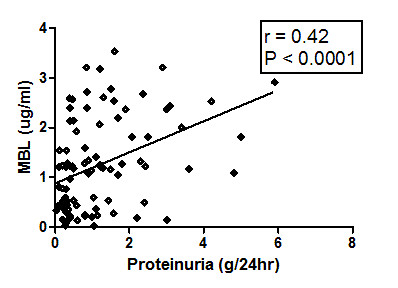
**Correlation of plasma mannose binding lectin (MBL) levels with proteinuria in female systemic lupus erythematosus (SLE) patients**. SLE patients (*n *= 93) were analyzed for association between MBL and proteinurea. Plasma MBL levels of SLE patients correlated positively with proteinurea. Dots represent individual samples. Correlation analysis was performed using the Spearman correlation coefficient. *P *< 0.05 was considered significant.

As shown in Figure 4, a positive correlation was observed between plasma MBL levels and 24-hr urinary protein (*P *< 0.0001, *r *= 0.42). However, there was no significant association between serum creatinine and MBL (data not shown).

## Discussion

MBL is a key molecule of the innate immune system that opsonises microbes through carbohydrate moieties and eliminates them through complement activation. Although the precise role of MBL in SLE pathogenesis is largely unknown, MBL plays a dual role in autoimmune disorders. Low levels of MBL impair the clearance of apoptotic bodies leading to production of antibody against self antigens, while increased MBL enhance activation of the complement system leading to the tissue damage observed in severe disease in SLE [18]. The present study demonstrates plasma MBL as a potential biomarker of SLE disease activity. Genetic aspects of MBL in SLE have been well investigated [19,29,30]. The variability in levels of serum MBL in lupus patients could be attributed to variants in the *MBL 2 *gene [31,32]. However, the reason for high MBL levels in SLE compared to healthy controls is not known. Some attribute it to an acute phase reactant consequent to ongoing inflammation [33]. However, literature is limited on the association of plasma MBL and disease activity. A study on Taiwanese pediatric SLE patients showed a positive correlation. In addition, plasma MBL also correlated positively with anti-dsDNA but not with complement factors (C3 and C4) [14]. A Japanese study of 147 adult patients did not conclusively demonstrate an association between MBL levels and disease activity, although serum MBL concentration did show significant association with serum C3 or CH_50 _levels [25]. Our study of 93 patients with lupus and 67 controls showed elevated plasma MBL in SLE compared to healthy controls corroborating the observations made in previous studies [14-16]. Interestingly, SLE patients also displayed higher plasma MBL levels compared to healthy controls with similar *MBL2 *genetic background [25]. Earlier reports have shown association of low-producer MBL genotypes with susceptibility to SLE [20,32,34-36]. The discordance in observation has not been satisfactorily explained, and further highlights the complexity associated with the pathogenesis of SLE. The level of serum MBL in SLE patients can be variable depending upon the equilibrium between production and consumption [25]. It stands to reason that the serum MBL values will vary depending on the time of sample collection. In our study, samples were collected from treatment-naive patients at the time of assessment. The distribution of plasma MBL in SLE patients (Figure 1A) was found to be variable with a mean level as low as 0.33ug/ml to a high 2.04 ug/ml. The levels correlated with specific clinical categories. Those with arbitrarily defined high and intermediate levels had major organ involvement, such as nephritis, in combination with carditis or serositis, and/or NPSLE. Most patients with musculoskeletal and cutaneous manifestations had low MBL.

Identification of a sensitive disease activity biomarker is essential in SLE management. Currently available biomarkers for disease activity are yet to be validated [37]. However, markers like anti-dsDNA, C3 and C4 are being widely used in clinical practice [38-41]. In the present study, plasma MBL levels correlated significantly with disease activity scores (SLEDAI). In addition, it correlated positively with anti-dsDNA and inversely with complement factors (C3 and C4). Complements play a pivotal role in the pathogenesis of SLE. They help opsonize and transport the immune complex, and maintain immune complex solubility by preventing formation of large insoluble aggregates [42]. Their role is further highlighted by increased predisposition to develop SLE in persons with deficiency of early complement components. The failure to clear apoptotic bodies containing nuclear components leads to a break in self tolerance and the development of SLE [43]. On the contrary, increased activation of complements mediates tissue damage. Low levels of C3 and C4 often correlate with disease flare [44]. MBL is intimately associated with complement activation through the lectin pathway, resulting in low complement levels in patients with high MBL.

Anti-dsDNA antibodies are found in 60% of patients with SLE. They strongly correlate with renal involvement but are of limited value for other manifestations of disease. Interestingly, the levels may fall at the peak of disease activity, probably due to deposition of antibodies in tissue [45]. In the current study we observed a positive correlation between MBL and anti-dsDNA antibodies. Tissue damage resulting from high levels of anti-dsDNA antibodies and MBL are distinct. Anti-ds DNA containing immune complex are deposited in tissue, resulting in tissue damage through the classical complement pathway [46], while MBL activates the lectin pathway of complement and causes tissue damage. Both appear to be independent variables of disease activity.

Lupus nephritis, a severe manifestation of SLE, affects nearly 50% of patients in the course of the disease [47], and 56% of the SLE patients in our series had nephritis. There are multiple mechanisms involved in the development of lupus nephritis. In the current study, proteinuria correlated positively with MBL levels (*P *< 0.0001). Patients with nephritis and other systemic involvement showed significantly higher levels of MBL (Table 2). Tissue damage in lupus nephritis is mediated by immune complexes or by binding of antibody to *in vivo *antigen, resulting in activation of the complement system and release of inflammatory cytokines. Previous studies have demonstrated deposition of MBL on glomerular tissue in SLE patients [48]. The possible ligand for MBL in the kidney is G0 glycoprotein of IgG [49]. Additionally, a significant galactose deficiency of IgG is also seen in lupus [50], which facilitates binding to MBL. Results from the current and previous studies indicate that nephritis may not directly correlate with plasma, but with tissue MBL levels.

## Conclusions

Plasma MBL levels are elevated in SLE patients and significantly correlate with various parameters of disease activity. Furthermore, they also correlate with increased proteinuria, indicating a possible role for MBL in lupus nephritis. MBL appears to be a promising biomarker in assessing SLE disease activity.

## Abbreviations

ACR: American College of Rheumatology; AIHA: autoimmune hemolytic anemia; C3: complement component 3; C4: complement component 4; ELISA: enzyme-linked immunosorbent assay; HC: healthy controls; IgG: immunoglobulin G; MBL: mannose binding lectin; NPSLE: neuropsychiatric systemic lupus erythematosus; SLE: systemic lupus erythematosus; SLEDAI: systemic lupus erythematosus disease activity index.

## Competing interests

The authors declare that they have no competing interests.

## Authors' contributions

AKP contributed to the design, performed the ELISA, data interpretation and writing of the first draft of the manuscript. JRP and SSP were involved in sample collection, data management and clinical categorization of samples. RT performed all routine tests. RT, BR and BKD contributed to the design, data interpretation, work supervision and critical revision of the manuscript. All authors read and approved the manuscript.
